# Types, Risk Factors, Consequences, and Detection Methods of Train Driver Fatigue and Distraction

**DOI:** 10.1155/2022/8328077

**Published:** 2022-03-24

**Authors:** Chaojie Fan, Shufang Huang, Shuxiang Lin, Diya Xu, Yong Peng, Shengen Yi

**Affiliations:** ^1^Key Laboratory of Traffic Safety on Track of Ministry of Education, School of Traffic and Transportation Engineering, Central South University, Changsha, China; ^2^Joint International Research Laboratory of Key Technology for Rail Traffic Safety, Central South University, Changsha, China; ^3^Hunan Industry Polytechnic, Changsha, China; ^4^Research Laboratory of Hepatobiliary Diseases General Surgical Department, The Second Xiangya Hospital, Central South University, Changsha, China

## Abstract

Train drivers' inattention, including fatigue and distraction, impairs their ability to drive and is the major risk factor for human-caused train accidents. Many experts have undertaken numerous studies on train driver exhaustion and distraction, but a systematic study is still missing. Through a systematic review, this work aims to outline the types, risk factors, consequences, and detection methods of train driver fatigue and distraction. The effects of central nervous fatigue and cognitive distraction in train drivers during driving are caused by rest and sleep schedules, workload, automation levels, and mobile phones. Furthermore, train drivers' fatigue and distraction can cause loss of concentration and slow reaction, resulting in dangerous driving behaviour such as speeding and SPAD. Researchers have combined subjective reporting, physiological parameters, and physical factors to construct detection algorithms with good results to detect train driver fatigue and distraction. This review offers recommendations for researchers looking into train driver fatigue and distraction. And it can also make valuable recommendations for future studies about railway traffic safety.

## 1. Introduction

The fatigue and distraction of train drivers directly influence their attention, cognitive ability, and judgment. Most railway accidents are caused by improper performance of drivers [1], which is called human factor-related accidents. Fatigue is another important factor leading to train accidents. According to an investigation of the Federal Railway Administration (FRA), in accidents caused by train drivers, fatigue ratio is 30–40% [2]. In the survey of Rail Accidents Investigation Branch (RAIB), 21% of train accidents were caused by driver fatigue, such as the derailment of a freight train in Melton Mowbray in 2006 and freight train collision at Leigh-on-Sea in 2008 [3]. Chang and Ju proposed statistics on the accident rate of passenger and freight trains in different cumulative driving hours in Taiwan from 1996 to 2006. They pointed out that the risk of accidents increased with increasing driving time, and the risk doubled after continuous driving for 4 hours [4]. The distraction growing anxiety and multitasking loads results in more signals being passed at danger (SPAD) [5, 6]. In September 2008, a rail accident occurred in Chatsworth, USA, causing 25 deaths. This accident was due to the driver's distraction caused by the mobile phone and then mistakenly passed the red signal light [7].  Table 1 shows the railway-performance shaping factor (R-PSF) analysis of 479 railway accidents in Europe; driver fatigue and distraction rank first among the causes of major accidents [8]. The investigation from Baysari et al. also confirms this result [9]. The results of 40 railway safety investigation reports show that nearly half of railway accidents were caused by equipment failure, while in the other cases, the most common causes of events were decreased alertness or decreased attention caused by fatigue (basic skill errors). In conclusion, fatigue and distraction are important causes of accidents in railways, resulting in more significant economic losses [10–12] and casualties.

There are a number of related research works and review papers about driver fatigue and distraction in the road traffic field [13–15]. However, train driver fatigue and distractions that may cause serious accidents, and extensive human and property damage have not received enough attention. This study aims to provide an in-depth review of the types, reasons, consequences, and detection methods of train driver fatigue and distraction by analysing the relevant peer-reviewed papers or reports.

The remainder of the paper is organized as follows: Sections 2to 5 briefly explain the types, reasons, consequences, and detection methods of train driver fatigue and distraction. Finally, Section 6 concludes this study.

## 2. Types of Train Driver Fatigue and Distraction

### 2.1. Fatigue Type of Train Driver

Fatigue is a complex state manifested by the lack of mental alertness, reduced physiological functions, and drowsiness [16]. Driving fatigue distracts the driver, increases operational errors, decreases information perception, processing judgment, and operational ability, and leads to microsleep as well as drowsiness. Researchers generally classify driving fatigue as central nervous fatigue, psychological fatigue, and physical fatigue according to the causes of its generation [13].

Central nervous fatigue is when the muscles are not working at high intensity while the nervous system is under high tension for long periods. It is due to monotonous and tedious work, resulting in decreased function and a state of inhibition of neural activity in the brain. During long hours of driving, the train drivers constantly receive and process external information, and always face various emergencies, which make nerves in a highly tense state. Therefore, their brain nerves are very active, and the brain loads are large. In addition, long-term monotonous driving will reduce the stimulation of the driver, and thus reduces the driver's alertness, delaysthinking, and reduces memory, resulting in central nervous fatigue.

Mental fatigue is a kind of driving fatigue caused by psychological factors. It usually occurs in the early stages of driving. In this status, the drivers subjectively feel tired, lose interest in driving, and even feel bored and tired. Mental fatigue changes with mood swings are very obvious, and drivers who suffer from mental fatigue do not have a reduced ability to complete the driving task but rather lack the subjective will to complete the driving task.

Physical fatigue is the phenomenon of stiffness, numbness, and pain in the driver's body organs due to long hours of driving or high intensity driving. During prolonged or high-intensity driving, frequent driving operations lead to continuous muscle contraction and energy substances in the muscles. Furthermore, the production of metabolites such as lactic acid and carbon dioxide causes sluggish movement, stiffness, and even pain in the organs. Metabolites enter the bloodstream and circulate through the body to further stimulate the nerves in the brain to produce fatigue. In addition, a fixed driving position causes stiffness and discomfort in the driver's back.

Train drivers have their own unique driving characteristics. Highly automated train operations and fixed tracks allow train drivers to drive without frequent driving maneuvers. Therefore, it is clear that physical fatigue is not the primary driving fatigue state in the train driver population. In addition, train drivers are extremely professional and need to undergo extensive training before they are qualified to drive trains. Therefore, the probability of mental fatigue is low. Train driving is a typical monotonous driving scenario; the train driver needs to lookout operation for a long time. The train track has a strong consistency and monotony, and the train driving route is long and time consuming, which makes the train central nervous fatigue unable to be avoided.

### 2.2. Distraction Type of Train Driver

Distraction is a mental state in which attention is not sufficiently directed and focused for the necessary time, or is completely diverted from what it should currently be directed and focused on to something unrelated, and distraction is reflected in driving behaviour, which is essentially dual-task driving. As defined by the National Highway Traffic Safety Administration, distractions can be classified into four categories according to their source: visual, auditory, biomechanical, and cognitive [17]: (1) Visual distractions, where the driver's visual range is obscured or the driver fails to perceive the road or losses visual acuity due to driver negligence. (2) Auditory distractions, where the driver concentrates on sounds (e.g., radio or passenger conversations) and ignores the road environment. (3) Biomechanical distractions (also called physical distractions), where the driver takes one or both hands off the steering wheel to operate other objects (e.g., using a cell phone) instead of concentrating on the physical tasks required for safe driving. (4) Cognitive distraction, which refers to the driver's energy being drawn to other things, thus reducing his or her reaction time and making the driver unable to complete road safety driving tasks.

For train drivers, the specificity of the train's route and the presence of real-time video monitoring systems ensure that the train driver needs to maintain a lookout posture and driving maneuvres at all times. The possibility of visual distraction and biomechanical distraction is extremely low. However, cognitive and auditory distractions are still inevitable.

## 3. Risk Factors of Train Driver Fatigue and Distraction

Filtness and Naweed [6, 18, 19] identified risk factors according to the industry documentation, accident reports, on-site observations, and discussions with train drivers. The results shown that poor sleep time and quality, shift work, high workload, inadequate recovery time and opportunity, and organizational factors are considered to be key factors influencing train driver fatigue. For driver distraction, nondriving-related factors such as mobile phones and driving-related factors such as the surrounding environment are often major causes of distraction for drivers.

### 3.1. Risk Factors of Train Driver Fatigue

#### 3.1.1. Rest and Sleep Time

Sleep or rest is often the primary method of relieving fatigue. A lack of sufficient sleep often makes train drivers more likely to feel fatigue during driving tasks. This is often due to subjective reasons on the part of the drivers themselves or objective reasons from the railway company. Poor sleep quality is unavoidable for some drivers, who suffer from the specific condition of sleep disorder (i.e., shift work sleep disorder (SWSD)) [20]. Drivers who lack the quality of sleep should be advised to move off from duty. In addition, family factors, excessive recreation, and irregularities in routine can also affect the quality and length of sleep of train drivers [18].

In most of the interviews with drivers, lack of sleep time was cited as a problem with the management system, particularly the shift work [21]. Train drivers often complain that shift times change frequently, and they have to adjust their work schedules frequently. The shift system is against the biological clock, with train drivers often having to drive at midnight, and the overly intensive shifts do not allow drivers to get enough rest. Rest periods tend to have a more pronounced effect on fatigue levels than the length of shift time. Iranian researchers divided 100 drivers into two groups, one performing long-mileage tasks and having more rest time, and one performing short-mileage tasks. The results showed that although the long-mileage drivers worked longer hours, the fatigue levels of the two groups were similar. This proves that sufficient rest time can compensate for the negative effects of long driving hours [22]. Obtaining eight hours of sleep is the only way to recover from the fatigue caused by a shift for train drivers [23]. A study based on a fatigue questionnaire showed that the risk of fatigue increased by 15% for every hour of shift time. What is more noteworthy is that the risk of severe sleepiness is 6 to 14 times higher for night shift drivers than for day shift drivers and approximately 2 times higher for early shift drivers [21]. In addition, there are significant individual differences in adaptation to shift work; managers often do not pay attention to the mental state of individual drivers, and the same shift system may not apply to all [24]. Moreover, drivers who were unmarried, had a college degree, and had limited driving experience showed lower fatigue control [18]. Managers should take these factors into account when scheduling to make sure that drivers have enough rest time.

#### 3.1.2. Workload

Train drivers' primary responsibility is to operate train, which takes up 50–63 percent of their shift [18]. The longest period of time spent in the cab was 2.5 hours [25]. The train driver should ensure that the train must be driven on the track safely, effectively, and on schedule [26–28]. During the whole driving task, train drivers are required to stay aware, perceive, interpret, recognize, anticipate and act on environmental signals in specific situations. Train drivers should have the ability to concentrate and to perform their work accurately. Selective, divided, and sustained attention (e.g., vigilance) is required. Train drivers should also have the ability to memorize relevant information. They must be capable of coping with emotional demands, low decision latitude, and a solitary work environment [26]. In addition to ensuring the safety of the train's normal operation, additional workloads such as assisting wheelchair boarding or increasing the number of platform stops often increase the risk of train driver fatigue. Therefore, the workload of train drivers is so heavy that it can easily cause fatigue.

#### 3.1.3. Automation Levels

To reduce the workload of train drivers and increase the capacity of the entire railroad carrier system without endangering safety, the level of railroad automation has been increasing with the development of autonomous driving technology. Railroad driving automation levels can be classified as GoA-0–GoA-4, ranging from manual to unattended.  Table 2 introduces the interpretation of the automation level. However, highly automated driving styles lead to new fatigue problems. The effect of task and workload on fatigue is formalized in active and passive fatigue theory [29]. According to this theory, persistently high workloads lead to active fatigue and persistently low workload situations lead to passive fatigue. In this sense, today's train drivers are likely to be affected by passive fatigue [30–33].

In the railroad sector, the sensitivity of train drivers to fatigue and the associated negative consequences have been confirmed by numerous studies in GoA-0 and GoA-1 [32, 34, 35]. Increasing the automation level from GoA-1 to GoA-2 profoundly changes the train driver's task (changing the role of the active manual train driver in GoA-0 and GoA-1 to that of a passive human observer of automated train operations in GoA-2) [36, 37]. However, workload levels in GoA-1 have been found to be in the underload range [38]. GoA-2 level technology introduces higher levels of automation into an already underloaded GoA-1, further reducing the workload and thus producing more pronounced passive fatigue and subsequent negative consequences [33, 39]. Thus, increasing the level of automation to GoA-2 may help to address capacity issues to some extent but does not appear to address task-induced fatigue and the associated negative consequences for operators. In contrast to GoA-2, the task was reconstructed in GoA-3 to free the train driver from constant visual monitoring (GoA-2) and instead specialize in handling specific and well-defined requests initiated by GoA-3 [40, 41]. Brandenburger et al. showed that participants in the GoA-3 group faced more activated task features in a more variable task environment than participants in the GoA-2 group. Thus, a lower severity of cognitive load deficit was evident from the higher workload scores. This resulted in less task-induced fatigue during the 2-hour shifts relative to the GoA-2 group. Skipping GoA-2 and moving directly from GoA-1 to GoA-3 is an excellent opportunity for rail operations to finally address chronic fatigue and increase capacity [40].

### 3.2. Risk Factors of Train Driver Distraction

Driver distraction is essentially due to the presence of a second task that interferes with the main task (driving task) [42]. In parts of the world where rail communications are not well developed such as South Africa, cell phones are an important tool for train drivers to communicate with other railroad workers and are used to compensate for the instability of radio communications [43]. However, the use of cell phones while driving has been shown to cause distraction and has also been shown to significantly reduce the visual search patterns used by drivers, reaction time, processes used for decision making, and the speed of the driver's ability to maintain [44]. The work environment can also induce distractions for railroad drivers, such as pedestrians along the tracks, animals crossing the railroad, or even large advertising signs. Although controlling the arrival time and speed of the train according to the dispatch center is considered a standardized procedure that train drivers must follow, repeatedly watching the dashboard and the clock undoubtedly increases the risk of distraction for train drivers, especially in the case of novice drivers. In addition, train drivers often experience cognitive distractions during monotonous driving, such as thinking about an upcoming wedding, a fun game, and the outcome of a ball game [6].

## 4. Consequences of Train Driver Fatigue and Distraction

In Section 1, we introduced driving accidents caused by driver fatigue and distracted states. In this section, we focus on analysing how fatigue and distraction affect driving behaviour, which leads to accidents. At present, the consequences of distraction and fatigue on train driver behaviour are mostly carried out by simulation or interviews.

Studies have confirmed that fatigue status affects people's attention, memory, vigilance, reaction time, and human coordination [45, 46], which further leads to accidents in railway. You et al. used an analog driving experiment to explore the relationship between driver's fatigue and behaviour. The results indicated that when the train driver is in the fatigue state, the operation accuracy was 126% lower than the normal state, and in the operation timeliness index, the time required for train drivers to respond and complete actions increased by 28.13% and 17.7%, respectively, compared with the normal state [47]. Other studies research train driver behaviour at different fatigue levels. With the increase of fatigue, drivers' psychomotor vigilance task (PVT) reaction time, extreme speed violations, subjective alertness, and penalty brake applications increased under moderate levels of fatigue, fuel use, draft (stretch) forces, breaking errors, and overbreaking increase. Under high levels of fatigue, the failure to act and maximum speed violations increase [48, 49]. In addition, there are differences in fatigue performance in sections with different track slopes [49, 50]. The study by Gregory et al. pointed out that fatigue and moderate drinking caused similar damages due to drivers' disengagement from the operating environment [51]. In a sleep deprivation experiment, lack of sleep caused an increase in fatigue score and affected the operation of the driver, with a 75% increase in the number of driving speed limit violations and a 55% increase in PVT indicators [52]. In the experimental report of driving distraction simulation, it is shown that with the growth of distraction level, different measures of operator performance decrease proportionally [53]. Filtness et al. conducted an interview survey among 22 railway drivers to study the impact of fatigue on driving and identified 5 different types of consequences caused by driver fatigue: SPAD, distraction, impaired judgment, train delay, and hiding fatigue state [18]. The consequences of driver inattention are summarized in Table 3.

## 5. Detection Methods of Train Driver Fatigue and Distraction

**Figure 1:**
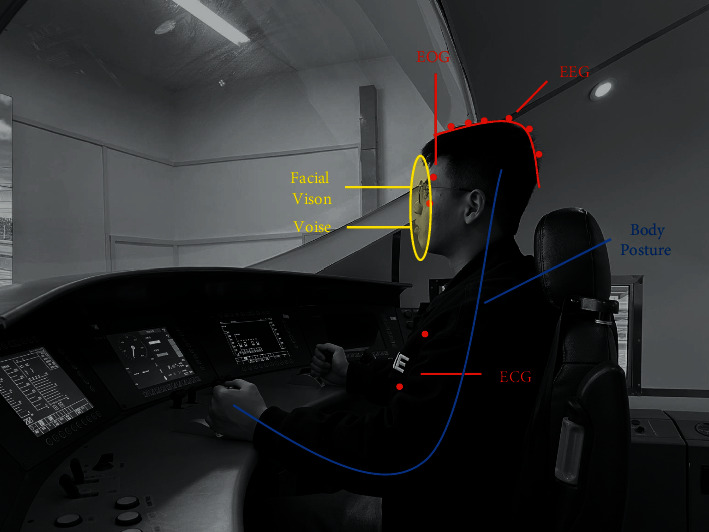
The detection method of train driver fatigue and distraction.

Train driver fatigue and distraction detection methods can be categorized based on input features into three categories: subjective reporting, biological features, and physical features.  Figure 1 shows the different placement positions of input features.

### 5.1. Subjective Reporting

Subjective reporting allows subjects to express their subjective feelings in a certain way, and they can be measured quantitatively or qualitatively [54]. In practice, subjective reportings are simple to use, easy for subjects to understand, less costly, more valid, and less disruptive to the driver's normal operation. In the field of train driver fatigue and distraction detection, Karolinska sleepiness scale (KSS) [55], stanford sleepiness scale (SSS) [56], and visual analogue scale to evaluate fatigue severity (VAS-F) [57] were used to detect the train driver fatigue. As shown in Table 4, both the KSS and SSS are the rating scores, the former with seven levels, and the latter with nine levels. Although subjective reports can directly capture drivers' perceptions of fatigue, drivers' estimates of their own fatigue are often inaccurate, exaggerated, or reduced. At the same time, different drivers have different fatigue perception abilities, so it is often necessary to normalize multiple subjective reports.

### 5.2. Biological Features

When drivers are fatigued, distracted, or under other poor driving conditions, the driver's biological signals are shifted from their normal state [58], and this shift is currently used to detect poor driving behaviour. Unlike other methods of detecting train driver fatigue using external features, the use of biological signals to detect fatigue is a more direct reflection of the driver's biological state. It has been shown that we can detect changes in the driver's state by biological signals at an early stage of fatigue. The biological signals can respond more quickly to changes in the driver's fatigue state with less delay than other methods [59]. Currently, researchers are using biological signals such as electroencephalography (EEG), electrocardiogram (ECG), and body temperature to detect fatigue in train drivers. The biological signal-based driver fatigue and distraction detection methods are shown in Table 5.

#### 5.2.1. Electroencephalography (EEG)

When the human brain is active, neurons transmit information to each other, resulting in weak electrical signals. EEG is a method of detecting these weak electrical signals to reflect the brain's activity [65]. Due to the unconcealable nature of brain activity, EEG can accurately detect a driver's inattention. Among the current biological signal-based inattention detection methods, EEG is considered the most promising. Many researchers have explored EEG signal-based inattention detection methods for train drivers. Torsvall et al. [60] studied the EEG changes in drivers driving at night. In the experiment, 11 train drivers were asked to complete two driving experiments on the same route, one during the day and one at night, for 4.5 hours each. At the end of the experiment, spectral analysis (FFT) of the EEG recordings showed a sharp increase in rated sleepiness during night travel, suggesting that train drivers may experience severe sleepiness during night work. Jap et al. [35] proposed several EEG discriminators of fatigue states by studying the changes in the EEG activity of train drivers during monotonous train driving. With the recent development of artificial intelligence technology, many researchers have applied machine learning algorithms to EEG analysis to identify driver fatigue. Using a wireless EEG acquisition device, Zhou et al. [62] collected EEG data from 10 train drivers and tested them on EEG, achieving a 99.4% correct classification rate within a 9-second time window. Zhai et al. [61] proposed a two-layer superimposed ensemble learning model based on EEG signals to estimate the alertness of highway drivers. The mean absolute error (MAE), root mean square error (RMSE), and goodness of fit (R-squared) are 70.14 (± 13.02) ms, 102.19 (± 22.18) ms, and 0.74 (± 0.09) for the estimated reaction time, respectively. Fan et al. [63] collected EEG signals from an EEG recording device placed on the driver's forehead and extracted many features from the EEG signals, including energy, entropy, rhythm-energy ratio, and frontal asymmetry ratio, and proposed a time-series ensemble learning method for detecting the fatigue state of train drivers. This study is the first to detect train driver fatigue and distraction simultaneously. However, most EEG-based driver inattention detection devices are currently only in the laboratory stage due to the complexity of the equipment required for EEG acquisition and for safety reasons. The design of inattention experiments in the laboratory and the criteria for inattention assessment are also still under discussion. There is also very little research on train drivers compared to cars. In the future, we need to design more convenient and comfortable EEG acquisition devices and lower latency EEG acquisition and processing paths to make this method suitable for practical applications.

#### 5.2.2. Electrocardiogram (ECG)

The periodic activity of cardiomyocytes in the body results in a potential difference at the body surface, a bioelectric change known as ECG. Heart rate and heart rate variability are the main ECG features currently associated with poor driving behaviour, such as fatigue and distraction. Heart rate is the number of heartbeats in a person at rest, and heart rate variability is a slight increase or decrease in the clockwise heart rate over a continuous cycle. Wilson et al. [66] showed that heart rate variability has a high correlation with fatigue and that the heart rate signal reflects the person's workload. Kalsbeek et al. [67] found that heart rate variability was significantly reduced when fatigue was present. Myrtek et al. [68] conducted an experiment with 12 high-speed train drivers and 11 mountain train drivers. By measuring the heart rate variability of these drivers, it was found that the train drivers were exposed to a higher mental load when the train started and slowed down and that the high-speed train drivers were at greater risk of monotonous driving-induced distractions. Gulhane et al. [69] devised an algorithm to indirectly estimate train driver fatigue using heart rate and heart rate variability as well as temperature differences between the inside and outside of the vehicle, and developed a hardware device to detect train driver fatigue. Ma et al. [64] used electrocardiogram (ECG) signals and eye movement features to determine the fatigue level of subjects and trained a nonlinear support vector machine (SVM) fatigue recognition model with a maximum recognition accuracy of 75%. ECG is noninvasive and portable. The equipment available for measuring ECG is relatively mature. However, there is little research on ECG in railway drivers, and the criteria to identify poor driving behaviour such as fatigue and distraction have neither been established nor is it clear how ECG can be integrated with other methods to assess driver fatigue and distraction. There is still a need to explore more correlations between ECG and driver fatigue and establish suitable identification models to apply ECG to practical applications.

### 5.3. Physical Features

In contrast to physiological signals, which require contact sensors, physical characteristics such as facial expressions, posture, and voice can often be captured by noncontact cameras, microphones, or even smartphones. As a result, physical characteristics have become the focus of research.

The blink rate, blink frequency, and average closed duration can all be utilized to identify weariness in a driver [4]. To evaluate driver weariness, numerous algorithms based on eye motivation have been developed. Yan et al. [17] used eye-movement data collected in a noncontact manner and after extracting the features of the data, different weight values were assigned to these features to reflect the primary and secondary relationships between the features. Finally, FWSVM was used to classify the driver's state, and the results showed an average accuracy of 90.98%, an average sensitivity of 92.01%, and an average specificity of 89.88%. Among all the eye-movement features, PERCLOS, which measures physiological weariness by the fraction of closed eyelids across time [6], is frequently utilized. The term PERCLOS is for “percent eye closure,” and the time with the eyes closed refers to a percentage of a given period. Gao et al. [70] conducted image acquisition of train driver faces using CCD image sensors. An AdaBoost classifier algorithm based on Haar features was used for face detection. Based on the detected faces, the eyes were located. The state of the eyes was detected within the face region using template matching to obtain two indicators for measuring the driver fatigue level. The first one is the ratio of human eye continuous closure time to a specific time (PERCLOS). And the second one is average human eye closure speed (AECS). Engineers of Guangzhou Railway Group Corporation proposed a novel mask-based method to find the eyes of train drivers in color pictures, and PERCLOS has been extracted to detect driving fatigue [71]. The experiments show that the algorithm is both effective and reliable with a 96.0% detection rate.

There is a strict set of gesture and slogan instructions for train drivers to ensure safety. Therefore, fatigue and distraction detection methods have received attention for train drivers' speech and gestures. Deep learning models were often widely used in such approaches [72–74]. Zhang et al. [75] built a voice fatigue database suitable for analysing the fatigue status of train crew members. A deep learning model based on voice phonemes was developed to detect driving fatigue. Liu et al. [76] developed the “urban rail driver gesture and mantra” operation combined with fatigue behaviour as a criterion to determine whether a driver is fatigued. The behaviour recognition module is based on a new dual-input 3DCNN model that is integrated into the Raspberry Pi. Zheng et al. [77] proposed a novel DBN-BPNN model in which the deep belief network (DBN) was used to extract feature set, and BPNN was the classifier. The average accuracy of this model can achieve 92.75%. For the mobile phone detection, a progressive calibration network (PCN) was used to define the detection area, and the CNN model finished a classification task to detect whether the area had a mobile phone [78].

There is no doubt that biometric identification methods have the advantages of being contactless, convenient, and low cost. However, there are limitations to physical feature-based identification methods for negative driving states where there is little change in physical characteristics, such as mental fatigue and cognitive distraction.

## 6. Conclusion

Train driver fatigue and distraction are undoubtedly crucial factors that jeopardize railway traffic safety, and the accidents result in significant property and human losses. This paper reviews and compares types, risk factors, consequences, and detection methods in the field of train driver fatigue and distraction. First, by analysing train drivers' driving tasks and driving environment, central nervous fatigue and cognitive distraction were identified as the most critical types of fatigue and distraction for train drivers. Second, it was summarized that the main risk factors for driver fatigue were rest and sleep time, workload, and automation level. At the same time, the use of mobile phones and the influence of the driving environment posed the risk of train driver distraction. Third, train driver fatigue and distraction often affect driver attention, reaction time, and memory capacity, and cause dangerous driving behaviours such as speeding and SPAD. Finally, the accurate detection of driver fatigue and distraction is critical in ensuring safety. Subjective reports, biological features, and physical features can all be used to build driver fatigue and distraction detection systems. Combining multimodal features may become a central research direction in the future.

## Figures and Tables

**Table 1:** Railway-performance shaping factors (R-PSFs) of train safety in accident analysis.

R-PSFs	Incidents	Accidents	Serious accidents	Total	Ratio (%)
Fatigue/distraction	154	187	71	352	21.00
Safety culture	89	115	80	284	16.95
Communication	130	107	47	284	16.95
Experience	137	70	25	232	13.84
System design	71	71	45	187	11.16
Quality of procedures	58	43	28	129	7.70
Perception	48	56	15	119	7.10
Pressure	30	22	6	58	3.46
Workload	17	7	7	31	1.85

**Table 2:** Automation levels.

Automation levels	Interpretation
GoA-0	Terminal operating system with manual driving
GoA-1	Nonautomatic train operation
GoA-2	Semiautomatic train operation
GoA-3	Driverless train operation
GoA-4	Unattended train operation

**Table 3:** Consequences of driver inattention.

Driver status	Indicators	Consequences
Fatigue	Operation accuracy	Decrease
	Response time	Increase
	Action time	
	PVT	
	Extreme speed	
	Subjective alertness	
	Penalty brake	
Distraction	Operator performance	Decrease

**Table 4:** Comparison of SSS and KSS.

Level	SSS	KSS
1	Feeling active, vital, alert, or wide awake	Extremely alert
2	Functioning at high levels, but not at peak; able to concentrate	Very alert
3	Awake, but relaxed; responsive but not fully alert	Alert
4	Somewhat foggy, let down	Rather alert
5	Foggy; losing interest in remaining awake; slowed down	Neither alert nor sleepy
6	Sleepy, woozy, fighting sleep; prefer to lie down	Some signs of sleepiness
7	No longer fighting sleep, sleep onset soon; having dream-like thoughts	Sleepy, but no effort to keep alert
8	—	Sleepy, some effort to keep alert
9	—	Very sleepy, fighting sleep

**Table 5:** Biological signal-based detection method.

Ref	Signal	Feature	Placement	Participants	Test environment	Method	Performance
[60]	EEG	PSD of *α*, *θ*, and *δ*	Head	11	Field test	Correlation Analysis	Significance
[35]	EEG	Band power, band ratio	Frontal temporal	50	Simulation	ANOVA	*P* < 0.05*P* < 0.01
[61]	EEG	PSD of *α*, *β*, *α*/*β*	Head	40	Simulation	SELM	MAE = 70.14 (± 13.02), RMSE = 102.19 (± 22.18), *R*2 = 0.74 (± 0.09)

[62]	EEG	PSD of *θ*, *α*, and *β*	Head	10	Simulation	RPCA	Accuracy = 99.4%
[63]	EEG	Energy, entropy, rhythmic, and asymmetry	Forehead	7	Simulation	RLX-TV	Accuracy_*F*_ = 96.0% Accuracy_*D*_ = 93.5%
[64]	ECG	HR, HRV	Chest	4	Simulation	SVM	Accuracy = 75%
